# Men’s conceptualization of gender-based violence directed to women in Alexandra Township, Johannesburg, South Africa

**DOI:** 10.1186/s12889-022-14616-5

**Published:** 2022-11-30

**Authors:** Kudakwashe Gracious Zinyemba, Khumbulani Hlongwana

**Affiliations:** 1 Discipline of Public Health Medicine, University of KwaZulu Natal, Durban, South Africa; 2 Cancer & Infectious Diseases Epidemiology Research Unit (CIDERU), College of Health Sciences, University of KwaZulu-Natal, Durban, South Africa

**Keywords:** Gender-based violence, Violence against women and girls, Intimate partner violence, Men’s perspectives, South Africa, Sub-Saharan Africa

## Abstract

**Background:**

Gender-based violence (GBV) is a crucial global public health challenge disenfranchising women and girls from enjoying their fundamental human rights, thereby threatening their well-being. While the concept of GBV does not imply that violence is always unidirectional, literature shows that women and girls are the most common victims of this type of violence. One in three women, globally, have suffered physical or sexual violence by an intimate partner or non-partner. Evidence has shown that a number of women who experience GBV varies widely, with 37% being in Eastern Mediterranean, 37.7% in South-East Asia, 29.8% in America, 36.6% in Africa, 44% in sub-Saharan Africa (SSA) and 53% in South Africa.

**Methods:**

Using a semi-structured face-to-face interview with fifteen conveniently sampled adult males, who met the selection criteria, this study explored men’s conceptualisation of GBV in Alexandra Township, using qualitative research methods.

**Results:**

Socio-economic factors and evolving cultural dynamics were perceived to be among the key factors aggravating gender-based violence. Participants viewed poverty and substance abuse as the main causes of violence towards women, a phenomenon tied to the growing frustration emanating from men’s inability to provide for their families. Cultural factors related to the patriarchal system and diminishing value of respect between men and women were identified as root causes of GBV. The participants also blamed the government for what they considered to be “too many rights” for women, resulting in men exerting their authority through abuse. Participants also expressed concerns over feminisation of GBV, asserting that men fall prey to GBV too. Partner infidelity and insecurities also contributed to GBV.

**Conclusion:**

The study results provided important insights on how men conceptualize GBV in Alexandra Township, South Africa. These results revealed that socio-economic conditions, alongside some gender stereotypes are pervasive and shape how men view GBV in Alexandra Township. This evidence is necessary for developing interventions aimed at curbing GBV and may also be suggestive of the need to redesign programmes targeting men, so that certain stereotypes can be uprooted.

## Background

Gender-based violence (GBV) is an important global public health challenge, which is deeply rooted in gender disparity and violation of human rights in all communities [1]. GBV affects fundamental human rights, thereby threatening the well-being of women and girls, worldwide [2]. While the concept of GBV does not imply that violence is always unidirectional, literature shows that women and girls are the most common victims of this type of violence [3–5]. GBV and violence against women and girls (VAWG) are terms that are often used interchangeably, with both frequently pointing at men as prime offenders and women and girls, as the victims [6]. Intimate partner violence (IPV) is the most common form of GBV, which includes physical or sexual abuse by an intimate partner or spouse [7]. However, the use of the term ‘gender-based’ has noteworthy implications, as it shows that various types of the VAWG are rooted in patriarchal gender norms and inequitable gender power [8].

The World Health Organisation (WHO), in collaboration with the London School of Hygiene and Tropical Medicine (LSHTM) and the South African Medical Research Council (SAMRC), analysed the secondary data from more than 80 countries, the results of which showed that 1 in 3 women, globally, have suffered physical or sexual violence by an intimate partner or non-partner sexual violence [9]. Evidence from the various regions has shown that a number of women who experience GBV varies widely, with 37% being in Eastern Mediterranean, 37.7% in South-East Asia, 29.8% in America, 36.6% in Africa, 44% in sub-Saharan Africa (SSA) and 53% in South Africa (SA) [10, 11].

Despite the worrying statistics of GBV, globally, the United Nations’ (UN) goal 5 of the 2030 Sustainable Development Goals (SDGs) sets an ambitious target of eradicating all forms of detrimental practices and VAWG by 2030 [12]. Approximately, 11.4 million women and girls are trafficked, globally, with more than 30% of women having experienced either physical violence, sexual violence or both [13]. According to Kusuma & Babu [14], at least 38.6% of all homicides recorded, globally, are likely to be perpetuated by intimate partners. In 2012, approximately 437 000 intentional homicide deaths were recorded, worldwide, with 95% of individuals convicted for the homicide being males [15]. Globally, at least 38% of women experience violence perpetrated by intimate partners [16].

In 2011, a dedicated population study on GBV directed to women was conducted in Gauteng province, South Africa, the results of which showed that 37.7% of women ever experienced physical or sexual intimate partner violence, 18.8% testified on sexual (IPV) and 46.2% experienced economic or emotional abuse from the sexual partner [17]. South Africa has a policy aimed at combating GBV, however evidence on its utility to address GBV, is scanty. According to the South Africa’s National Strategic Plan (NSP) on GBV and femicide, results from non-population-based studies are not generalisable to the country’s population [17]. Nevertheless, it is still of major concern that prevalence rates reported in other studies are constantly higher than 50%, with men invariably being leading perpetrators [17].

The 2017 Gauteng study revealed that only 1 in 23 women experiencing sexual abuse report to the police, and this is reflective of gross underreporting of sexual offences [18]. There is a reason to believe that the true reflection of sexual assaults is substantially higher than what is being reported to the police [19]. Of concern, is the fact that almost half (46%) of the victims of sexual assaults were children, suggesting that GBV is mainly perpetrated by those who are expected to provide protection [18]. The Report by an Independent Police Investigative Directorate (IPID) illustrated an increase in rape cases by police officers and a whopping 230% increase in sexual abuse cases perpetrated by teachers in the last 5 years [18]. In fact, the implication of police in sexual offences may be one of the contributors to gross underreporting of sexual crimes, as victims may not be convinced that police will act in their best interest.

Some factors that favour a great occurrence of gender-based violence are already mapped by other studies from a legal, socio-cultural, and socio-economic perspective. These factors include the payment of lobola, use of alcohol and drugs.

The legal and socio-cultural system works against the rights of women in that the patriarchal norms deny women the right to make decisions, thereby leaving women vulnerable to GBV [20]. Cultural practices, such as paying lobola (bride price), place men in a powerful position over women, often leaving women with little power in the marriage [21]. This assertion is supported by Chiweshe’s earlier study on Christian women’s experiences of IPV in Zimbabwe, which revealed that lobola had become the source of oppression and ultimately led to IPV [22]. The social norms associated with lobola, such as being submissive to the husband and sex as a conjugal right, has a silencing effect on women, forcing them to accept violence within marriages as they feel, by virtue of lobola payment, it is normal and should not be questioned [22].

There is a body of evidence suggesting that men feel some entitlement over their female partners once they have paid lobola. For example, a study conducted in Mpumalanga, Eastern Cape and Limpopo showed that 84% women interviewed agreed that as soon as he partakes in lobola payments, it is considered culturally appropriate for a man to physically abuse his wife, if she does something perceived to be immoral [23]. The practice that men have the right to abuse their women if they neglect household responsibilities or for infidelity, has been observed in other settings [24–26]. Omeje & Chikwedu [27] went on to say that women are not allowed to discuss their marital experiences with anyone, as these are regarded as family affairs, which should be resolved within their families. Regrettably, this arrangement further creates fertile ground for abuse to take place.

A cross-sectional study conducted among 1 568 male participants in four districts of Sri Lanka revealed that one-fourth of the participants considered sexual teasing harmless and a majority of the respondents were of the view that women deserve to be beaten at times, if they do not perform their supposedly primary responsibilities, such as taking care of the family and household [28]. The study further revealed men’s stereotyped view that a woman cannot decline sexual intercourse with her husband, and that she should make it her obligation to prevent falling pregnant. Various authors are in agreement in that inequitable power distribution across genders render women vulnerable to violence by men, in the name of discipline or maintenance of order in their home [29–31].

Alcohol and substance abuse also contribute to GBV, in that they directly affect the cognitive and physical function, leaving individuals less capable of negotiating non-violent ways to resolve disputes within relationships [32]. A study conducted in Cape Town, South Africa, found a strong association between violence and alcohol use, with heavy alcohol use leading to negative styles of resolving issues [33]. Furthermore, drinking with a girlfriend was found to increase the expectations of her agreeing to sexual activities, thereby resulting in the use of force in non-consensual circumstances [34]. These findings are consistent with Ngonga’s study linking alcohol consumption and IPV, with men often using alcohol as their excuse for perpetrating violence [32].

From Hidayat’s perspective in an Indonesian study, most men were concerned about the women’s attitudes, behaviours and responses, implying that they provoke men into committing GBV [32]. This perspective is supported by the results of a study conducted in South Africa, whereby one of the respondents believed that women wearing short skirts is a contributing factor to GBV [33]. Various participants linked men’s perpetration of GBV to women’s behaviours and clothing, regrettably sending a message that women should take the responsibility for the GBV and not men [33, 34]. Societal expectations of masculinities to men’s perpetration of violence are a phenomenon of research importance; hence this study explored men’s conceptualisation of GBV. Given that fingers often point to men as perpetrators of GBV, it is important to explore how they conceptualize GBV, so that appropriate interventional strategies can be proposed.

GBV endangers the well-being of women, globally, adding to the burden of disease [35] and imposing strain on women’s physical and emotional well-being. GBV has negative outcomes for girls and women, including sexually transmitted diseases, unwanted pregnancies, induced miscarriages and forced abortions [35–37]. A survey conducted in South Africa, showed that women in abusive relationships were 48% more likely to be infected with HIV than their non-abused female counterparts [37]. The Tanzanian study revealed that abrasion and bleeding caused by forced sexual intercourse exacerbated HIV transmission [37]. Gender inequality, male manipulative behaviour and intimate partner violence threats limit women’s ability to negotiate for consistent condom use, refuse unwanted sex and the ultimate risks of HIV infection [38]. Women who experience GBV do not only engage in HIV risky behaviours, including unprotected sex [39], but are also hindered from engaging in women’s HIV care [40, 41]. A systematic review and meta-analysis conducted by Hatcher and colleagues exploring the effects of IPV on antiretroviral (ART) use, showed that IPV was associated with lower ART uptake, lower adherence and lower chances of viral suppression [40], and these results are consistent with that of Leddy et al. [39] on the linkages between GBV, HIV and their effects on HIV care continuum.

Gender-based violence can have an intense and long-lasting effects on the survivor’s mental health, such as anxiety, increased peril of post-traumatic stress disorder and depression [42]. Rees et al. [43], confirmed that women exposed to GBV experience have high risk of mental disorders and suicidal behaviours. Nonetheless, Tsai [44] argued that there are ethical and methodological challenges in defining the chronological cycle of GBV and mental disorders. The author also revealed that it is not easy to conduct surveys inquiring on the mental health problems from the survivors or family members, because the abuse usually occurs within the confined family context [44].

A study conducted amongst students in Ethiopia confirmed that there was an association between GBV and mental health disorder, which often resulted in depression [45]. GBV is also associated with mood, anxiety and substance abuse [46]. A study conducted in rural KwaZulu-Natal, South Africa, on the correlation between GBV, psychosocial stress and self-esteem, revealed that the perpetrators also suffered from mental health, such as anxiety and depression, resulting from GBV perpetration [47]. The study also indicated that 60% of men were involved in psychologically abusing women, which affects the mental health of women [47].

The above shows that VAWG negatively affects the victims leading to unwanted pregnancies, greater propensity to be infected with HIV, low ART uptake, anxiety, and depression which adds to the burden of diseases.

GBV can be best understood through ecological theory as a methodological framework underpinning the study [32]. According to Heise’s ecological theory, nested, multilevel structural and normative systems cause multiple forms of violence against women [24]. Heise [24], further analysed the ecological theory as a framework made up of four stages of analysis, which are in the form of four interactive concentric circles referred to as systems of interactions, whereby each system is dependent on the environment of a particular person [33]. The four factors include individual, relationship, community and societal factors. This theory is useful for understanding the intricacies of gender-based violence, since it conceptualizes violence as a multifaceted phenomenon with various factors at play [34].Therefore, from the ecological perspective, there is no single factor that explains why some people are more violent than others, but the goal is to focus on the various ways through which gender-based violence manifests [48].

## Methods

### Study setting

The study was conducted in Alexandra Township, informally known as Alex, with locals often referring to it as Gomora. This Township is in Johannesburg, South Africa, the city known as the economic hub of the country. Alex is one of the poorest townships, predominantly made up of informal settlements in Johannesburg [49, 50].

### Study design

This study explored men’s conceptualisation of GBV in Alexandra Township, South Africa, using a simple exploratory qualitative design guided by the ecological framework.

### Characteristics of participants

The participants in this study were males over the age of 18 years residing in Alexandra Township. These males excluded those that had received any form of training on GBV from the non-governmental organisations operating in the area. This was aimed at ensuring that the perspectives provided by the participants were original and not influenced by prior training/s or programmes on GBV. All participants voluntarily participated in the study after the study had been well explained to them and subsequently signed informed consent forms.

### Participant selection

The participants were purposively selected using convenience sampling technique. The participants who met the selection criteria, easily accessible and available to be part of the study were selected. The recruitment took place in the public places, mainly on the streets near the participants’ property yards and the in-depth interviews were conducted in the participants’ preferred spaces, which consistently happened to be their property yards.

### Data generation and analysis

Data were generated through semi-structured face-to-face interviews, using interview guide, which allowed for follow-ups, probes, and spontaneous questions. While the interviews were conducted in English; some participants mixed English with their home languages (IsiZulu, Ndebele, and Tsonga). Semi-structured in-depth interviews were considered most suitable, given the exploratory nature of the study, to conversationally elicit personal and controversial issues on the research subject [51].

All interviews were audio-recorded (with participants’ permission). The researcher transcribed the English audiotapes in verbatim, whereas a research assistant was recruited to assist with transcribing and translating audiotapes from IsiZulu into English. Field notes were written during the interviews to complement the audio materials.

The transcribed data was analysed using thematic data analysis. It is argued that thematic analysis is a method that can be used within a range of epistemologies and research questions to identify, analyse, organize, describe, and report themes discovered within a data set [52]. It is also useful in highlighting similarities, differences and generating anticipated insights when examining the participants’ perspectives and producing a clear report [52].

A codebook was developed after reading all transcripts, as part of familiarisation with the data, a necessary step to qualitative data analysis. Given a relatively small sample size [15], manual coding was possible. The coding process also assisted in making sense of the data and identify themes and sub-themes. The data was then analysed into themes and sub-themes using the epistemology theoretical approach. Epistemology clarifies how researchers acquire the truth or knowledge, as it is a theory of knowledge of what can be known, and the criteria used to justify it being knowable [53].

### Ethical considerations

The study was approved by the Biomedical Research Ethics Committee (Ref. BREC/00001956/2020) of the University of KwaZulu-Natal. All potential participants were fully informed about the study prior to signing informed consent forms. Participation was strictly voluntary, informed consent was obtained from all the study participants, and the names used to reference their verbatim quotes are not real names, but pseudonyms.

## Results

The participants’ ranged from 24 to 49 years, with 27% being unemployed, while 40 and 33% were self-employed and employed, respectively (Table 1). More than half (53%) of the participants had high school level of education, and the remaining 47% had tertiary education. Almost half (47%) of the participants were single, with married and divorced participants constituting 40 and 13%, respectively (Table 1).

**Table 1:** Participants' demographics profiles

Characteristics	Number	%
**Age(years)**		
20–24	1	7
25–29	3	20
30–39	6	40
40–49	5	33
**Education status**		
High school	8	53
Tertiary	7	47
**Marital status**		
Single	7	47
Married	6	40
Divorced	2	13
**Employment status**		
Employed	5	33
Unemployed	4	27
Self employed	6	40
**Number of people living in the household**		
Adults		
1–3	5	33
4–9	10	67

Five themes and six sub-themes emerged from the analysis (Table 2) and these varied from intrapersonal to broader societal and systemic issues.

**Table 2:** Main themes and sub-themes from the analysis

Themes	Sub-themes
**Participants’ understanding of GBV complexities and directionality**	
**Interplay between socio-economic issues and GBV**	Poverty, unemployment and GBV
	Alcohol, drug abuse and GBV
**Evolving cultural issues and GBV**	Patriarchal system, the value of respect and GBV
	Culture of silence and GBV
**Insecurities, infidelity and GBV**	
**Perceived government’s women-centred approach to GBV interventions**	Women’s rights and GBV
	Inequitable protection of male GBV victims by law enforcement agencies

### Participants’ understanding of GBV complexities and directionality

There was a convergence of views among participants in that GBV is a huge problem, not only in Alexandra township, but in the whole of South Africa. Participants started sharing stories of the cases of GBV in their communities, some of which ending either in the arrest of offenders or even termination of relationships.

“The dangers of this [GBV] can be someone ending up being in jail, divorced or even murder, and suicide”- Mandla, 49-year-old unemployed divorced man.

Participants expressed that violence in Alexandra was complex and not only restricted to GBV, as xenophobic attacks were also pervasive.

“…Here it is a mixture [all kinds of violence] because you know Alex is well known for xenophobic attacks”-Mqhele, 29-year-old self-employed single man.

However, it was also pointed out that there are two-sides to these stories, meaning that GBV can emanate from either side.

“There are two sides. In those relationships people are not sustained by love for each other but there’s constant abuse. A woman is abusing a man and a man is abusing a woman”- Themba, 45-year-old self-employed married man.

Participants shared similar sentiments on their understanding of the magnitude of the GBV in South Africa, including what it means, as they used phrases, such as: “beating your partner or wife” (Thabo, Amo and Noah), “shouting” (Mpho), “smashing of belongings” (Mpho), “violation of rights” (Siya), “chopped-cut into pieces” (Qhawe) and “killing” (Themba and Langa) to describe the concept. None of the participants based their conceptualisation of GBV to their personal experiences, but they conceded that their sharing was from their personal observations.

A view that GBV should not be treated as a problem only for women permeated throughout the interviews, with a suggestion that some men are also being abused by women.

“…It is not a one-sided matter; it exists on both genders and women abuse men. I personally would like if men abuse can also be acknowledged as an issue that the men in the society are facing. Women should also be jailed because we witness women abusing men”- Themba, 45-year-old self-employed married man.

Participants were in agreement in viewing GBV as a deplorable act that makes men look like monsters, given the resultant loss of lives for of innocent children, thereby destroying families and hurting others.

“It hurts me because l am not happy seeing people we love being killed in this manner, what hurts me even more is when men are angry, they kill even the children”-Themba, 45-year-old self-employed married man.

“I am very crossed, l feel bad about GBV because many families are destroyed. The father ends up being a monster killing his children and wife”-Langa, 46-year-old self-employed married man*.*

#### Interplay between socio-economic issues and GBV

##### Poverty, unemployment and GBV

Participants identified poor living conditions evident through acoustically weak and congested housing structures as something that makes it impossible for them not to notice when GBV is happening in their neighbours. Others contended that they have observed GBV happening within their communities in Alexandra, as supported by the following statement:

“The living arrangement like our shacks are in close proximity to each other, so it just makes it almost impossible for me not to hear the upsets of my neighbours because as you know when there is violence people scream sometimes”-Mpho, 30-year-old unemployed single man.

Participants identified poverty and unemployment as intricately linked to the occurrence of GBV in Alexandra, with men arguably resorting to GBV due to frustrations related to their inability to provide for their families. For example, men get frustrated and physically abusive if women ask for basic needs that they cannot provide. In support of this assertion, one participant shared the following:

“I feel poverty contributes [to GBV] like for example, a man is coming from work, and he is tired when he gets home. Maybe the wife has not cooked because cooking oil is finished, and she points out to the man that cooking oil is finished and maybe he may just respond in a rude way and the woman is also not having it because I mean she just simply point out cooking oil is finished…an argument will start between the two and it can end into a physical fight”- Thabo, 35-year-old employed single man.

“…But what I observed is causing gender-based violence is due to us being unemployed especially as men, women are fed up with us sitting at home and just doing nothing, so that’s where the gender-based violence come from”- Teddy, 35-year-old self-employed married man.

Some men abuse their partners because if women are the ones who are working, they are not willing to take care of the men, thereby resulting in men resorting to the use of physical power to have their demands met.

“You find that a woman earns more, or the man is jobless…that working woman will not be taking care of the man and as a result there is financial abuse perpetrated by the woman because of this historical thing that they should be provided for and not be providers”-Mandla, 49-year-old unemployed divorced man.

However, participants also shared sentiments that women who are financially dependent to men, are more vulnerable to GBV as compared to women who are independent, because they can easily leave the abusive relationship.

“Women should learn to be independent so that they can stand on their own so that when a man becomes violent, she can move and start another life. Some force the situation of staying with a violent man because she has no means of making her own survival”-Langa, 46-year old self employed married man.

### Alcohol, drug abuse and GBV

Participants shared that people living in Alexandra consume a lot of alcohol in order to, presumably, forget about their miserable lives. Easy access to alcohol and drugs in the Alexandra community, as every corner has taverns and bars, exacerbates GBV. Substances are used not only as social events, but also these intoxicating substances were thought to relieve unemployment and poverty-related stress.

“It is mainly poverty, alcohol and drug abuse because in these slums a lot of people are abusing alcohol and drugs, maybe to hide from their miseries, hide from poverty”- Mandla, 49-year-old unemployed divorced man.

“Everywhere you go there is a tavern, but say you live in Sandton [neighbouring upmarket place] like on a Sunday today the establishments are closed and there are no taverns, even if you want a beer you cannot access it, but in these slums, beer is available everywhere even at 3am beer is avaialable, drugs all over”- Mandla, 49-year-old unemployed divorced man.

“Men here in Alexandra are excessive drinkers, they surrender their lives into drinking. They get drunk with alcohol, dagga and high with nyaope, they just love being intoxivated”- Langa, 46-year-old self-employed married man.

Participants asserted that, as a result of alcohol and drug abuse, both women and men do not know how to communicate with each other when intoxicated. Participants further shared that the violence erupts when women ask the men not to spend too much money on alcohol and when both parties are drunk. Participants also shared that GBV can start when a woman does not concede to the man’s demands for money to buy alcohol or drugs, especially given, arguably, low men’s reasoning capacity when drunk.

“…in Alexandra, the only option is to drink alcohol, everyone drinks and when they are drunk there is a misunderstanding in communication. A drunk person versus a drunk person, noise starts from there and that is why we have gender-based violence”-Noah, 42-year old employed divorced man.

“It is such conditions [alcohol and drug abuse] that lead to gender-based violence, like I am demanding money from either my mother or my wife to buy drugs. We end up exchanging words and abusing my wife or my mother. The issue is that I want to get my fix and I know she has money, and when I ask for it she does not give me because she has other uses for it”-John, 28-year old self employed married man.

Another perspective to how alcohol contributes to GBV related to bad advices menprovided to each other when they are gathered for drinking. These include the beating of ones wife if she is supposedly being disrespectful or as part of discipline, thereby instilling fear.

“Some men just want to instill fear in women because at times, it is something that you actually hear men talking about over beer”- Thabo, 35-year-old employed single man.

Participants were asked to explain the link between high alcohol intake, high unemployement and poverty in the community. The participants shared that you do not always need to have money everyday in order to get drunk, men in Alex do not mind sharing alcohol and whoever has money that day will buy and others will drink and also buy when they have money. They are united as a community and understand that times can be tough.

“…People are so united in a way that even if you do not have money, you will end up getting drunk. I mean if you go to the tavern, you can actually see people sharing a quart of beer maybe three or four will be sitting there and buying a quart of beer, if it finishes then buy another, so you might find out from those four people maybe one person have money but everyone gets drunk”- Thabo, 35-year-old employed single man.

“…And the money they are drinking, they do not have it. You find that they took the money from the wife or they are being given by friends”- Melusi, 32-year-old employed married man.

#### Evolving cultural issues and GBV

##### Culture of silence and GBVsec3

Participants asserted that the “mind your own business” pervasive in Alexandra has made it difficult for people to report the observed GBV in their neighbourhoods, especially given the sensitive nature of GBV. Thabo explained:

“…issues involving two people in a marriage like in an African culture there is a saying that ïndaba zabantu ababili azingenwa [issues involving two people in romantic relationship cannot be interfered with]”- Thabo, 35-year-old employed single man.

The community’s inability to confront the perpetrators is influenced by their status in the community and the prevailing weapons, which threatens the safety of those intending to intervene.

“…People are exposed to weapons in South Africa, it is not easy to speak about it [GBV], even if it happens to people you know because it gets scary to address certain things depending on how feared you are in the community”- Stompie, 28-year-old employed single man.

Children are particularly disadvantaged on issues of GBV because they are culturally not allowed to interfere with the issues between the parents as explained by Mpho:

“Like growing up in an African or family setup, you are raised not to raise your concern even if what [GBV] is happening between your parents does not sit well with you”- Mpho, 30-year-old unemployed single man.

Evolution of African culture to adapt the western culture, was seen as an enabler of GBV, as the western culture purportedly weakened family structures, thereby allowing GBV to thrive. Mandla explained the following:

“Cultural background plays a role because there is no home without quarrels, but our family backgrounds will help us for example our uncles, extended families will come forward to resolve our issues before they turn into something bigger [GBV]. My take is family structure has to be revised because black people, I think they are taking foreign cultures out of hand and disintegrating the culture they used to have”- Mandla, 49-year-old unemployed divorced man.

### Patriarchal system, the value of respect and GBV

Some participants viewed patriarchy as an inadvertent cause of GBV, with some perpetrating GBV without even realising it, either due to lack of awareness or they feel justified through the use of customary belief system, as Siya shared:

“Sometimes men do violence against women without realising that they’re violating the rights of a woman”-45-year-old, unemployed married man.

Participants identified women’s non-recognition of men as the head of the household as a violation of cultural norms, thereby contributing to GBV in Alexandra. Men also feel they need to protect patriarchal system in order to preserve their superiority to women.

“The problem in Alexandra’s household is that anyone can be a household leader, children and everyone are leaders, hence there is no order in Alexandra”-Themba, 45-year old self employed married man.

“The other problem is that they [women] get drunk, they get drunk like men, they no longer have any respect to bear the responsibility of taking care of the family as women”-Langa, 46-year old self employed married man.

“It is cultural again, you know our culture informs us men as head and women just have to be submissive”-Mpho, 30-year old unemployed single man.

Participants felt that woman make unreasonable demands that men cannot fulfil, yet they do not respect men and alcohol abuse by women contribute to the lack of respect. Participants further pointed out that in Alex, there is no culture and women and children do not respect men as the leader of the house.

“…So we grew up knowing that a man is the head of the house and when ladies are drunk they start disrespecting men and it ends up physical”-Noah, 46-year old employed divorced man.

Men feel that since they propose to the women, women should respect their rules as the husband. One participant shared the following:

“I request we have respect here in the house, if l am talking to you as my wife, l request you show me respect because l first proposed to you, you never proposed to me”-Themba, 45-year old self employed married man*.*

#### Insecurities, infidelity and GBV

Participants shared that another contributing factor to GBV is their insecurities or the women being unfailthful. A fight can start when a man checks his wife’s cellphone or receives a call from another man, the man will become suspicious..

“It is more of the insecurities on the men’s side of things because in most cases they think their women are up to something with other people and they start getting a little bit violent”-Amo, 31-year old unemployed single man.

Participants also raised the cheating by women in relationships as another trigger for GBV.

“He burns the entire family all because maybe he found out his wife was cheating on him”- Langa, 46-year old self employed married man.

“Sometimes people do things outside of their relationship, so it ends up being violent because men sometimes do not know how to handle that type of a situation. Instead of walking away or maybe try sit and find out what is happening, they rather take it upon themselves and start being violent trying to fix the situation”- Amo, 31-year old unemployed single man.

#### Government’s women-centred approach to GBV interventions

##### Women’s rights and GBV

The participants were not pleased with how the government is handling GBV, as they felt women are being given “too many rights” which are making them not respect men. Participants also shared that women can start the physical fight, but men ends up arrested, thorugh the manipulation women-friendly systems.

“Lack of respect is the main instigator for the reason that women take advantage of the fact that the South African law defends and supports them”-Themba, 45-year old self employed married man*.*

### Inequitable protection of male GBV victims by law enforcement agencies

Consequently, men feel they do not enjoy the same protection as women.

“The government interfered with the rights of men and today we no longer have rights”-Themba, 45-year old self employed married man.

“ We as man have no one who is protecting us but women, the government protects them”-Noah, 42-year old employed divorced man.

## Discussion

Participants shared similar understanding of what constitutes GBV and used phrases like “beating your partner”, smashing of belongings”, “violation and rights” and “killings”. The ecological framework offered useful insights into the understanding of multi-layered and multidimensionaldrivers of GBV, at least from the perspectives of males. The study revealed that men are generally not only aware of the occurrence of GBV as a social-ill engulfing the communities, but are also concerned about its deadly repercussions in families and communities, alike. Socio-economic conditions, evolving cultural dynamics, partner insecurities and infedility, as well as government’s women-centred approach to GBV, were all perceived to be among the key factors aggravating GBV in the community. Socio-economic factors and related social ills, such as poverty, unemployment, alcohol and drug abuse were all identified as important issues necessary for a broader understanding of GBV. Poverty and unemployment were particularly linked with the growing frustration experienced by men, thereby resulting in increased GBV, especially since men find themselves being unable to provide for their families. Participants’ use of the concept poverty was not confined to unemployment, but included those whose incomes could not meet their basic needs.

Socio-economic difficulties have ripple-effect on increasing alcohol and drug abuse, which are also linked to gender based violence. However, results noted alcohol and drug abuse as something pervasive across gender, although intoxicated men were seen as likely to resort to abuse than their female counterparts. Easy access to alcohol and drugs in the study setting jeorpadise efforts to curb GBV. Men participating in this study also got into finger-pointing by alleging that women do provoke them, something that results in physical abuse, a phenomenon that arguably goes unreported to law enforcement authorities until it gets worse. Partner infedility and insecurities were also identified as important factors to GBV. However, the participants did not share how infidelity on the part of a male partner affects GBV. Participants of the study blamed women-centred government policies as contributing factors to ever-increasing GBV, as well as weak protection of male GBV victims by law enforcement agencies.

The results of this study identified men’s frustration emanating from lack of employment as a contributory factor to GBV and this is congruent with a study conducted in the European asylum reception facilities, which also made similar findings [54]. The ripple-effect of unemployment on men’s ability to meet the basic needs for their families perpetuated GBV [55], as was the case in the study by Mosavel et al. [56] conducted in South Africa. Shiva et al. [57] have linked alcohol, drug abuse and GBV, especially since communication mannerism gets lost when people are intoxicated, a phenomenon that is supported by our findings. Despite the association between alcohol and GBV being established, the pathway for this association requires further research [57].

Gender-based violence has many contributing factors in SA that can be traced back to cultural and traditional practices, gender inequalities and discrimination in all forms, such as economic, social, religious or political aspects [19]. These factors are rooted in unequal power relations between women and men, low status of women in communities and beliefs that men are superior and entitled to certain things, including toxic masculinity [30]. Culture as a key factor on GBV was supported by the findings of Le Mat et al. [55] study, which indicated that cultural factors are usually used as an excuse for violent behaviours and this is consistent with our findings. The study participants in our study asserted that, as the head of the house, they expected respect from their female partners. A study conducted in KwaZulu-Natal revealed that some men belonging to the Zulu nation used culture to justify their patriarchal practices [58] and culture has generally been used to justify power imbalances often resulting in GBV [59], a phenomenon that is continuosly reinforced by the patriarchal value system. This system identifies women as inferior to men, with illiteracy, poverty and low status of women being prevalent in the communities [41]. Economic liberation enables women to escape abusive relationships better than economically dependent women [52]. When women are capable of providing for themselves, own assets and have control to their resources, they tend to have more economic power and they can use it to escape abusive relationships [53]. Participants shared that women should also learn to be independent, so that they do not rely on men who may manipulate their dependancy to abuse them. Sadly, violence is not only confined to home settings, as some women escape violence from home, only to encounter it in other locations, such as work, markets and public spaces, owing to social and gender inequalities and societal norms [53, 54]. Participants from our own study also demanded that they should be respected on account of being heads of the households. The patriarchal attitudes frequently favour men over women and there is an acknowledgement that GBV is largely caused by the interplay between individual, societal and cultural factors interacting at different levels of communities [42]. There is documented evidence linking infedility to GBV, 37% of the participants indicated that infidelity contributes to GBV [60], this is caused when a partner is suspected of being unfaithful and misplaced anger [61]. Respondents from this study also drew a link between infedility, jealousy and GBV, which is often a result of a partner checking each other’s cellphone. However, participants in our study were silent on infidelity committed by men, unsurprisingly so, as they were all males. The study setting also have a culture of not intervening on GBV issues, as community members view GBV as an internal affair that can only be handled by the couple or family concerned.

Applying the ecological framework to the understanding of our findings places the role played by societal, community, relationship and individual factors to perpetuating GBV in context. The different factors influence the occurance of GBV, for example the study showed that societal factors like cultural norms in a community with a high unemployment rate influence alcoholism, which, in turn exacerbate GBV. This illustrates the intedepencance of these factors to perpetuating GBV in communities.

### Strengths

The study explored how men in Alexandra conceptualize GBV from their own perspective. The study participants were recruited from the community, thereby making our observations and other data more dynamic than those generated from captive environments. This study makes an important contribution to the body of evidence in South Africa on GBV from a men’s perspectives, since most studies are female-focused.

### Limitations

The researcher’s limited familiarity with the setting did not place her in good standing for recruiting information-rich participants that would help us deepen our understanding of the phenomenon. The fact that the researcher was a young female who interviewed males may have rendered the study prone to social desirability bias. However, this was mitigated through detailed explanation of the purpose of the study and the value of truthfulness. While efforts were made to properly document the study processes, transferability of findings may have been affected by the weaknesses pervasive in sampling and recruitment strategy.

### Contribution of evidence-based research

There is paucity of data in South Africa on how men conceptualize GBV. The study findings can be used to guide the government and stakeholders on the design strategies aimed at reducing GBV.

## Conclusion

The study results provided important insights on how men conceptualize GBV in Alexandra Township, South Africa. These results revealed that socio-economic conditions, culture, and patriachy alongside some gender stereoptypes are pervasive and shape how men view GBV in Alexandra Township. The socio-cultural system works against the rights of women and the patriarchal norms leaves women vulnerable to GBV. While there is large body of evidence on GBV in South Africa broadly, there is paucity of data/evidence on how men conceptualise gender-based violence, hence this study was aimed at developing these data using qualitative research methods.This evidence is necessary for developing interventions aimed at curbing GBV and may also be suggestive of the need to redesign programmes targeting men, so that certain stereotypes can be uprooted.

## Data Availability

The datasets generated and/or analysed during the current study are not publicly available due to the fact that they are part of an ongoing academic (for degree) study but are available from the corresponding author on reasonable request.

## Abbreviations

GBV: Gender-Based Violence
HIV: Human-Immunodeficiency Virus
IPV: Intimate Partner Violence
LSHTM: London School of Hygiene and Tropical Medicine
SAMRC: South African Medical Research Council
UN: United Nations
WHO: World Health Organisation
